# Determinants of default to fully completion of immunization among children aged 12 to 23 months in south Ethiopia: unmatched case-control study

**DOI:** 10.11604/pamj.2016.23.100.7879

**Published:** 2016-03-16

**Authors:** Abiyot Getachew Asfaw, Digsu Negese Koye, Amsalu Feleke Demssie, Ejigu Gebeye Zeleke, Yalemzewod Assefa Gelaw

**Affiliations:** 1 Wolayta Zone Central Statistics Bureau, Southern, Ethiopia; 2 University of Gondar, College of Medicine and Health Sciences, Institute of Public Health, Ethiopia

**Keywords:** Default to immunization, unmatched case-control, children, South Ethiopia

## Abstract

**Introduction:**

Immunization is a cost effective interventions of vaccine preventable disease. There is still, 2.5 million children die by vaccine preventable disease every year in developing countries. In Ethiopia, default to fully completion of child immunization is high and determinants of default to completions are not explored well in the study setting. The aim of the study was to identify determinants of default to fully completion of immunization among children between ages 12 to 23 months in Sodo Zurea District, Southern Ethiopia.

**Methods:**

Community based unmatched case-control study was conducted. Census was done to identify cases and controls before the actual data collection. A total of 344 samples (172 cases and 172 controls) were selected by simple random sampling technique. Cases were children in the age group of 12 to 23 months old who missed at least one dose from the recommended schedule. Bivariable and multivariable binary logistic regression was used to identify the determinant factors. Odds ratio, 95%CI and p - value less than 0.05 was used to measure the presence and strength of the association.

**Results:**

Mothers of infants who are unable to read and write (AOR=8.9; 95%CI: 2.4, 33.9) and attended primary school (AOR=4.1; 95% CI:1.4-15.8), mothers who had no postnatal care follow up (AOR=0.4; 95%CI: 0.3, 0.7), good maternal knowledge towards immunization (AOR= 0.5; 95% CI: 0.3, 0.8) and maternal favorable perception towards uses of health institution for maternal and child care (AOR= 0.2; 95% CI: 0.1, 0.6) were significant determinant factors to default to fully completion of immunization.

**Conclusion:**

Working on maternal education, postnatal care follow up, promoting maternal knowledge and perception about child immunization are recommended measures to mitigate defaults to complete immunization.

## Introduction

Immunization has become the most effective public health measure for the control of infectious vaccine preventable disease and one of the best tools to decrease child mortality and morbidity [1]. Despite their impact, vaccines have generally received less attention than drugs, but the vaccine landscape is shifting, and new opportunities, challenges, and debates have pushed vaccines to the center of global health discussions [2]. Incomplete immunization can put children at greater risk of acquiring a disease and partial immunization coverage against vaccine preventable diseases is a significant public health challenge in developing countries [3]. Immunization averts an estimated 2 to 3 million deaths every year in all age groups from diphtheria, tetanus, pertussis (whooping cough) and measles worldwide. Africa and Southeast Asia are regions most affected by the problem of vaccine preventable disease of which more than 70% of these children live in 10 developing countries and South Africa including Ethiopia [4]. According to Medicines Sans Frontiers (MSF) Access Campaign-Oxfam 2010 Vaccine - Report, there are multiple suggested factors that make delivering vaccines to children in developing countries makes difficult; lack of research and promotion for better-adapted and needed vaccines, and fragile health systems with corresponding health personnel shortages [2]. Vaccination is one of the indicators used to monitor progress towards the achievement of Millennium Development Goal (MDG) goal 4, i.e. the reduction of child morbidity and mortality [5]. Ethiopia has been implementing different strategies by considering it as one of the most cost-effective measures to reach 90% coverage at the end of the implementation of the program. However, the national vaccination coverage still remains at 24% in 2011with huge variation among regions and place of residence within the country [5–7]. Previous studies done in Ethiopia [8, 9] indicated that the main factors affecting to complete immunization are multiple such as age of the mother, parents education, knowledge about immunization, antenatal care (ANC) and postnatal care (PNC), marital status, mother's occupation, sex of the child, birth place, birth order, place of residence and distance to health intuitions. Even though there is a consensus about the above problems to the contribution of low coverage of vaccination, the problem still persisted with much little improvement posing a challenge to the country health care delivery system [5, 6, 8]. Therefore studying the determinants of incomplete immunization allows for effective implementation of policy where there is a high rate of defaulter.

## Methods

A community based unmatched case-control study was done from April 15 to 30, 2013 in Sodo Zurea District, South Ethiopia. Sodo Zurea District is found in Wolayta Zone, Southern Ethiopia, and 329 km far from Addis Ababa, capital of Ethiopia. The District has 4 urban and 33 rural kebeles (small sub units). A stratified sampling technique was used to select the study participants. The district was classified in to two strata; urban and rural. Then two urban and three rural kebeles were randomly selected. For the purpose of this study, census was done to identify the eligible mothers which was start from north side of the kebele goes by serpentine motion to end of total proportionally identified households. During the census, cases and controls are identified based on self report of care takers (mothers) and/or observations of immunization card. Cases were children with the age of 12 to 23 months old that miss at least one dose from a total of 8 vaccines within 23 months old. Controls were children in the age interval of 12 to 23 months old and completed the recommended dose of vaccines according to the schedules of immunization. By using the census registration number as a sampling frame participants were selected via table of random generation number. The sample size was calculated using EPI INFO software version 7 Statcal program via unmatched case control formula; considering proportion of illiterate mothers or caretakers of among cases 36.6% and 27.8% among controls [8], a 5% significance level, power of 80%, 1:1 ratio of case to control and 10% non - response rate. The sample size was estimated to be 344 (172 cases and 172 controls). Data were collected via interview with a pre-tested and structured questionnaire. Data collectors and supervisors were health professionals. Training was given to the data collectors and supervisors on the objective, confidentiality of information, respondent's right and techniques of interview prior to data collection. Data on immunization was collected either by immunization card or mothers/caretakers verbal report. The information on the doses and types of vaccines was collected from the card and (or) based on mothers (caretakers) report on the history of the child to categorized case and control.

Maternal knowledge towards immunization was assessed by asking seven knowledge related questions. A score of 1 was given if the care takers answered the given questions correctly and 0 if not. The sum of each questions were computed their mean and categorized in to two groups ((> mean as good knowledge and ≤ Mean as poor knowledge)) [8]. The returned questionnaires were checked for completeness by the data collectors, supervisors and investigator before data entry. The data were entered into Epi INFO™7 and exported to SPSS version 20 for further data cleaning and further analysis. Descriptive statistics were done to summarize the data in relation to the different variables. Binary logistic regression model was used to identify the determinant variables. Variables having P - value ≤0.2 in the bi-variable analysis were entered into multivariate logistic regression models to control the effect of confounding. Both bivariable and multivariable logistic regression models were used to identify the determinant factors of default to fully complete immunization. Odds ratio (OR) with 95% confidence interval (CI) were calculated to measure the strength of associations. Variables when p - value ≤ 0.05 was consider as statistically significant factors associated.

This study was carried out after getting ethical approval from the Institutional Review Committee of Institute of Public Health, University of Gondar. Before the ethical approval, the proposal was provided to reviewers to assure the ethical issues. Finally, the ethical review committee approved the verbal consent by considering that the research has not serious harm to the study participants. Before the interview and measurements, the interviewer fully explained the purpose of the study to each participant and obtained full verbal informed consent from each study participant. They were also informed that participation was on voluntary basis and they can withdraw at any time if they are not comfortable about the interview.

## Results

**Socio -demographic characteristics of mothers**: a total of 344 mothers of infants (172 cases and 172 controls) were interviewed. Among cases, 52 (30.2%) were from urban areas. Of respondents, the majority of mothers were married 320(93%), protestant followers 170 (52%), unable to read and write 201 (58.4%) and house wife (91%) by occupation. More than half 191(55.6%) of infants were male (Table 1).

**Table 1:** Socio-demographic characteristics of mothers of infants in Sodo Zurea District, Wolayta Zone, SNNPR, Southern Ethiopia, April 2013 (n=344)

Study Variables	Category	Default to complete immunization	
		Cases no. (%)	Controls no. (%)
**Religion**	Orthodox	76 (44.2%)	83 (48.3%)
	Protestant	93 (54.1%)	85 (49.4%)
	Others	3 (1.7%)	4 (2.3%)
**Educational status**	Not read and write	118 (68.6%)	83 (48.3%)
	Primary school	51 (29.7%)	70 (40.7%)
	Secondary and above	3 (1.7%)	19 (11.0%)
**Ethnicity**	Wolayta	171(100%)	171(99.4%)
	Amhara	0 (0.0%)	1 (0.6%)
**Marital Status**	Single	2 (1.2%)	0 (0.0%)
	Marred	155 (90.1%)	165 (95.9%)
	Divorced	6 (3.5%)	2 (1.2%)
	Separated	6 (3.5%)	3 (1.7%)
	Widowed	3 (1.7%)	2 (1.2%)
**Family size**	≤4	59 (34.3%)	63 (36.6%)
	≥5	113 (65.7%)	109 (63.4%)
**Total children alive**	1	28 (16.3%)	23 (13.4%)
	2-4	104 (60.5%)	101(58.7%)
	≥5	40 (23.2%)	48 (27.9%)
**Sex of a child**	Male	97(56.7%)	94 (54.7%)
	Female	75 (43.6)	78 (45.3)
**Birth order**	≤2	54 (31.4%)	57(33.1%)
	3-4	69 (40.1%)	58 (33.8%)
	>4	49 (28.5%)	57(33.1%)
**Place of delivery**	Home	165 (95.9%)	164(95.3%)
	Health institution	7(4.1%)	8 (4.7%)
**Vaccination Card availability**	Yes	67(39.0%)	104 (60.5%)
	No	105 (61.0%)	68 (39.5%)
**Mothers occupation**	House wife	151(87.8%)	162(94.2%)
	Merchant	15 (8.7%)	9 (5.2%)
	Daily laborer	6 (3.5%)	1(0.6%)
Monthly Income (ETB) *	<500	132 (76.7%)	107(62.2%)
	501-1000	39 (22.7%)	62 (36.1%)
	>1000	1(0.6%)	3 (1.7%)
**Previous child death**	Yes	66 (38.4%)	87(50.6%)
	No	106 (61.6%)	85(49.4%)

* ETB Ethiopian Birr

**Determinants of default to complete immunization**: variables such as, educational status of caregiver, child death history, number of family size, and total number of children ever born, perceived side effect of vaccination, ANC, PNC, maternal knowledge towards immunization, and perception towards health services, time taken to reach to the health facilities (accessibility) were independent determinants of default to fully completion of immunization at a p - value ≤ 0.2. Whereas, education status of care givers, PNC, maternal knowledge towards immunization, perception towards health services were statistically significant with default to complete immunization in multivariable analysis at a p - value ≤ 0.05. Infants born from mothers who unable to read and write [AOR=8.9, (95% CI: 2.4, 33.9)] and had primary education [AOR=4.1, (95%CI: 1.1, 15.8)] by educational status were 9times and 4 times more likely to default to complete immunization when compared to infants born from mothers who had secondary and above, respectively. Infants who were born from mothers that had PNC follow up were 60% [AOR=0.4, (95% CI: 0.3, 0.7)] less likely to default to complete immunization compared to infants who were born from mothers who didn't have PNC follow up. Infants born from mothers who had good knowledge towards immunization were 50% [AOR=0.5, (95% CI; 0.3, 0.8) less likely to default to complete immunization as compared to infants born from mothers who had poor knowledge towards immunization. Regarding perception towards uses of health institution for maternal and child health service, respondents who have good perception towards uses of health institution were 80% less likely to default complete immunization [AOR= 0.2, (95%CI: 0.1, 0.6)] as compared to mothers who had not good perception (Table 2).

**Table 2:** Multivariate analysis of determinants of default to complete immunization among children 12 to 23 months old, Sodo Zurea District, SNNPR, southern Ethiopia, April 2013 (n=344)

Study Variables (category)	Default to complete immunization		Crude OR (95%CI)	Adjusted OR (95%CI)
	Cases	Controls		
**Education status**				
Un able to read and write	118 (68.6%)	83 (48.3%)	9.0 (2.6, 31.4)*	8.9 (2.4, 33.9)*
Primary school	51 (29.7%)	70(40.7%)	4.61(1.3, 16.4)*	4.1 (1.1,15.8)*
Secondary and above	3 (1.7%)	19 (11.0%)	1.0	1.0
**Family size**				
≤4	59 (34.3%)	63(36.6%)	0.9 (0.6, 1.4)	
≥5	113 (65.7%)	109 (63.4%)	1.0	
**Total children ever born**				
≤4	120 (69.8%)	111 (64.5%)	1.3 (0.8, 2.0)	
≥5	52 (30.2%)	61(35.5%)	1.0	
**Knowledge towards immunization**				
Yes	36 (20.9%)	73(42.4%)	0.4 (0.2, 0.6)	0.5 (0.3, 0.8)*
No	136 (79.1%)	99 (57.6%)	1.0	1.0
**Perceived side effect**				
Yes	13 (7.6%)	4(2.3%)	3.4 (1.1, 10.8)	
No	159 (92.4%)	168 (97.7%)	1.0	
**History of child death**				
Yes	66 (38.4%)	87(50.6%)	0.6 (0.4, 0.9)*	
No	106 (61.6%)	85(49.4%)	1.0	
**ANC follow up**				
Yes	124(72.1%)	147(85.5%)	0.4 (0.3, 0.8)	
No	48 (27.9%)	25(14.5%)	1.0	
**PNC follow up**				
Yes	55(32.0%)	96 (55.8%)	0.4 (0.2, 0.6)*	0.4 (0.3, 0.7)*
No	117(68.0%)	76 (44.2%)	1.0	1.0
**Perception towards health institution visit**				
Good	157(91.3%)	169 (98.3%)	0.2 (0.1, 0.7)	0.2 (0.1, 0.6)*
Not good	15 (8.7%)	3 (1.7%)	1.0	1.0
**Time taken to reach health institution in minutes**				
<15	27(15.7%)	12 (7.0%)	0.4 (0.2,0.8)*	
15-30	61(35.5%)	64 (37.2%)	0.42 (0.2, 0.9)*	
31-60	84 (48.8%)	96 (55.8%)	1.0	

* **=** p value less than 0.05

## Discussion

Immunization is one of the most powerful and cost-effective of all health interventions. It prevents debilitating illness and disability, and saves millions of lives every year around the world. Vaccines have the power not only to save, but also to transform, lives - giving children a chance to grow up healthy, go to school, and improve their life prospects [9, 10]. Coherent with the previous studies [8, 10–14]; educational status of the mothers is the determinant factors to complete fully immunization in the present study. This is due to the fact that educated mothers could have better knowledge and decision making skill on different issues of the child health including timing on immunizing their children as compared to illiterate mothers. Children whose mothers had PNC follow up after birth had decreased their chance by 60% to default to complete immunization than mothers who didn't follow up of PNC which is supported with the previous study carried out in Ethiopia [15]. This is due to the fact that mothers who had PNC follow up could appreciate the advantage of timely and fully completed immunization children for the development of their infants.

This study also revealed mothers who had good knowledge towards immunization were 50% less likely to default to complete immunization in line with the previous studies done in Ethiopia [15, 16]. This is may be due to the fact that mothers’ who had good knowledge towards immunization are not be a house wife's employee and return back to their job before completing the immunization schedule of their infants. In Ethiopia maternity leave for governmental employee is only three months that could have been also the reason to default to complete their infant's immunization.

Moreover, mothers of infants with good perception towards uses of health service were 80% less likely to default to complete immunization than those mothers who had unfavorable perception towards uses of health service. This finding is in agreement with previous studies conducted in Ethiopia [8, 15]. The explanation related to inaccessibility of health institutions and inadequate health professionals may be the possible cause to default to complete. The potential limitation of our study was first, recall bias where mothers may difficult to remember the immunization status of their children, total doses of vaccine the child took and misclassification of cases and controls. Second, the study was not supported by qualitative study to explore reasons of mothers/caretakers to default to complete.

## Conclusion

The result of this study provided variables such as; un able to read and write and attended primary school in educational status, had not postnatal follow up, maternal knowledge towards immunization, maternal perception towards uses of health service were independent predictors of default to complete immunization. Improve maternal education; promote health education, implementation of health institution counseling and support and strengthening postnatal care services is recommended as inevitable measure to curb poor immunization practice in the study setting.

### What is known about this topic

- Fully vaccination coverage is still below the goal in Ethiopia;
- Children of mothers living in urban areas, children of mothers with at least some educational status, children of mothers with some occupation;
- Mothers who had male children and children of mothers in the highest wealth quintile were more likely to fully completed immunization within the target age groups of a child.

### What this study adds

- This study adds as a determinant factors of fully completed child vaccination the following variables;
- Mothers had PNC follow up, good perceptions towards visiting health institution and had knowledge on immunization were more likely to fully completed immunization;
- This study also support mothers of children who had good knowledge on vaccine preventable disease with favorable health seeking behavior helps to achieve fully vaccination coverage goal.
